# Vitamin A Supplementation Programs and Country-Level Evidence of Vitamin A Deficiency

**DOI:** 10.3390/nu9030190

**Published:** 2017-02-24

**Authors:** James P. Wirth, Nicolai Petry, Sherry A. Tanumihardjo, Lisa M. Rogers, Erin McLean, Alison Greig, Greg S. Garrett, Rolf D. W. Klemm, Fabian Rohner

**Affiliations:** 1 GroundWork, 7306 Fläsch, Switzerland; nico@groundworkhealth.org (N.P.); fabian@groundworkhealth.org (F.R.); 2 Department of Nutritional Sciences, University of Wisconsin-Madison, Madison, WI 53706, USA; sherry@nutrisci.wisc.edu; 3 Department of Nutrition for Health and Development, World Health Organization, 1207 Geneva, Switzerland; rogersl@who.int; 4 UNICEF Headquarters, New York, NY 10017, USA; emclean@unicef.org; 5 Infant and Young Child Nutrition, Micronutrient Initiative, Ottawa, ON K2P 2K3, Canada; agreig@micronutrient.org; 6 Global Alliance for Improved Nutrition, 1202 Geneva, Switzerland; ggarrett@gainhealth.org; 7 Helen Keller International, New York, NY 10010, USA; rklemm@hki.org; 8 Johns Hopkins Bloomberg School of Public Health, Baltimore, MD 21205, USA

**Keywords:** vitamin A, deficiency, supplementation, fortification, biofortification, MNPs, programs

## Abstract

Vitamin A supplementation (VAS) programs targeted at children aged 6–59 months are implemented in many countries. By improving immune function, vitamin A (VA) reduces mortality associated with measles, diarrhea, and other illnesses. There is currently a debate regarding the relevance of VAS, but amidst the debate, researchers acknowledge that the majority of nationally-representative data on VA status is outdated. To address this data gap and contribute to the debate, we examined data from 82 countries implementing VAS programs, identified other VA programs, and assessed the recentness of national VA deficiency (VAD) data. We found that two-thirds of the countries explored either have no VAD data or data that were >10 years old (i.e., measured before 2006), which included twenty countries with VAS coverage ≥70%. Fifty-one VAS programs were implemented in parallel with at least one other VA intervention, and of these, 27 countries either had no VAD data or data collected in 2005 or earlier. To fill these gaps in VAD data, countries implementing VAS and other VA interventions should measure VA status in children at least every 10 years. At the same time, the coverage of VA interventions can also be measured. We identified three countries that have scaled down VAS, but given the lack of VA deficiency data, this would be a premature undertaking in most countries without appropriate status assessment. While the global debate about VAS is important, more attention should be directed towards individual countries where programmatic decisions are made.

## 1. Introduction

Vitamin A deficiency (VAD) is considered one of the most prevalent micronutrient deficiencies worldwide, mainly affecting children in developing countries [1]. It is estimated that globally about 30% of children <5 years of age are vitamin A (VA) deficient, and about 2% of all deaths are attributable to VAD in this age group [2]. VAD is also a major cause of preventable childhood blindness [1]. The transfer of VA in breast milk from the mother to the child depends on the status of the mother, and thus VAD often develops early in life [3,4], particularly in populations that consume diets low in provitamin A carotenoids and/or populations prone to infections, which often lead to reduced intake or depletion of VA stores [5,6,7].

Supplementation with high doses of preformed VA is currently one of the most widely-used interventions delivering VA. At present, more than 80 countries worldwide are implementing universal VA supplementation (VAS) programs targeted to children 6–59 months of age through semi-annual national campaigns. Due to VA’s influence on immune function, supplementation with a high-dose of VA is designed to reduce mortality associated with measles, diarrhea, and other illnesses [8] and not to sustainably improve the VA status of populations. A high-dose of VA improves VA status for only up to three months in children who have low dietary intake [9]. For this reason, while VAS provides a protective dose in the presence of VAD, complementary interventions are needed for VAD control such as VA (bio-) fortification, micronutrient powders, dietary diversity, nutrition education, and prevention and control of infectious disease.

VAS programs began in the 1990s in response to evidence demonstrating the association between VAD and increased childhood mortality [10,11]. Between 1990 and 2013, more than 40 efficacy studies of VAS in children 6–59 months of age were conducted, and two systematic reviews and meta-analyses have concluded that VA supplements can considerably reduce mortality and morbidity during childhood [12,13].

In 2013, following the publication of these reviews, Awasti et al. [14] published results of a large cluster-randomized effectiveness trial in India (the Deworming and Enhanced Vitamin A (DEVTA) trial) that showed, conversely, that semi-annual VAS did not reduce mortality. In 2014, Fisker et al. [15] reported the results of a randomized controlled trial in Guinea-Bissau, and concluded that “VAS had no overall effect on mortality but was associated with reduced mortality in girls and increased mortality in boys”. In 2015, Mason et al. [16] published a policy paper, referring to the results of the Awasti study and suggesting that VAS programs are “less relevant” because of a decreasing prevalence of measles and diarrhea and the fact that VA supplements do not impact the underlying VAD. Moreover, they suggested a “policy shift” away from VAS towards other interventions (e.g., fortification) that would sustainably reduce VAD. To date, the World Health Organization (WHO) continues to support biannual supplementation to children 6–59 months of age in settings where VAD is a public health problem, and recommends that “VAS should be used along with other strategies to improve vitamin A intakes, such as dietary diversity and food fortification” [17].

In response, the article by Mason et al., several researchers [18,19,20] directly disputed their proposed policy shift. In brief, researchers identified multiple design flaws of the DEVTA trial and cited repeated criticisms of the DEVTA trial’s methods and conclusions [18], attributed near elimination of severe VAD, xerophthalmia, and childhood blindness to VAS [19], and stated that more studies are needed before any phase out of VAS can be conducted [20]. In line with the Global Alliance for Vitamin A (GAVA) recommendations [21], these researchers [18] also suggested that countries should only consider scaling back VAS programs in populations where there is evidence that VAD is no longer a public health problem (i.e., <5% prevalence of VAD). Researchers on both sides of this debate in the literature have acknowledged that more data on VA status are needed so that countries can make informed programmatic decisions. Mason et al. [16] suggest “...increasing regular VA intakes, while monitoring VAD changes”, and Bhutta and Baker [19] advocate for an “...increased quality, frequency and disaggregation of measurement of vitamin A deficiency” and of effective coverage of VAS programs. Moreover, Stevens et al., who examined global trends in VAD from 1993 to 2013 [2] and reported a 10% reduction in the global VAD prevalence (from 39% in 1993 to 29% in 2013), noted that the majority of data on VA status are outdated, and the available data make it difficult to accurately estimate the global prevalence of VAD.

To address this data gap and contribute to the debate about VAS, we examined countries with a routine VAS program and within these countries identified nationally-representative VA status surveys. We identified the most recent survey to determine if data are current or outdated. Amongst these countries, we also examined the presence of VA fortification and biofortification, and micronutrient powder programs. By identifying concretely what data exist in each country, and what programs exist alongside VAS, we aim to assist national programmers and other stakeholders to identify their place in the wider debate. What countries need data, or more-current data, to better implement and target their VAS program? Where do data show that VAS should be scaled up? Where should it be scaled back based on recent data and other programs in place?

## 2. Materials and Methods

### 2.1. VAS Programs

We used the United Nation’s Children’s Fund’s (UNICEF) State of the World’s Children (SOWC) VAS coverage database [22] updated in November 2015, which reports supplementation coverage between 2000 and 2014 to identify countries with possible VAS programs. The set of countries included in the VAS coverage database was updated in 2014, and includes countries that had: (i) mortality rates among children under-5 years of age (U5MR) >70 deaths per 100,000 population in the year 2000 as per estimates released by the Inter-agency Group for Child Mortality Estimation (UN IGME) in September 2013; and/or (ii) a history of national level VAS programming; and/or (iii) a severe public health problem of VAD in the year 2000 or earlier, defined according to WHO recommended cut offs for declaring VAD a public health problem; or (iv) a history of national level VAS programming combined with a moderate or mild public health problem of VAD around the year 2000 defined according to WHO cut offs. The data related to VAD were based on nationally representative data from the grey literature as well as data in the WHO Micronutrients Database [23].

From UNICEF’s database, we used the most recent estimates of the proportion of children receiving two consecutive doses of VA supplements per annum. The proportion of children 6–59 months of age that were fully protected with two annual doses in one calendar year is estimated by taking the lower of the two annual semester-wise coverage estimates. This employs an assumption that children who received a dose in the semester with the lower coverage were also reached in the semester with higher coverage. In cases where the VAS in both semesters was only delivered through events (i.e., no VAS was delivered through routine contacts with the health system), if the timing between doses was less than four or more than eight months apart, annual two dose coverage is estimated to be zero.

### 2.2. Vitamin A Fortification, Bio-Fortification, and Micronutrient Powder Programs

Countries implementing VA programs were identified from multiple sources. The Global Alliance for Improved Nutrition’s (GAIN) 2015 *Fortifying our Future* report [24] and website maps [25] were used to identify countries implementing mandatory and voluntary fortification programs where VA is added to vegetable oil. VA-fortified sugar programs were identified in two separate publications: the 2006 WHO food fortification guidelines [26] and a review article listing all food fortification programs in Africa [27]. VA-fortified wheat flour and maize flour programs were identified in a publication by Klemm et al. [28] and country profiles on the Food Fortification Initiative website [29]. A 2011 global review of home fortification interventions [30] and preliminary results from UNICEF’s Nutridash platform for 2015 [31] were used to identify national and sub-national micronutrient powder (MNP) programs. MNP pilot and emergency programs were not included in our analysis. HarvestPlus’ 2015 global biofortified crop map [32] was used to identify all countries that have *released* crops (e.g., cassava, maize, and/or sweet potato) biofortified with provitamin A carotenoids. Countries currently testing biofortified crops were not included as part of our review.

### 2.3. Search Strategy for Vitamin A Deficiency Data and Inclusion and Exclusion Criteria

For countries identified in the UNICEF SOWC database, we first explored the WHO Micronutrients Database for nationally-representative surveys measuring VAD amongst children 6–59 months of age. We then conducted a literature search in June 2016 using Web of Science, Google Scholar and PubMed to identify other VA status surveys. Keywords used were: (vitamin A) OR (serum retinol) OR (plasma retinol) OR (retinol binding protein) AND (country name). We also conducted searches of the websites of WHO, UNICEF, GAIN, and the Micronutrient Initiative to identify unpublished surveys. Lastly, the co-authors used their respective organizational networks to identify any surveys not identified using the previous search approaches. We included nationally-representative surveys of children 6–59 months of age as the primary target age range, but were flexible by including surveys with slightly older children and children with a smaller age range (e.g., children 6–35 months in Liberia). We excluded surveys and studies that: (a) were representative of smaller-administrative areas (e.g., provinces, cities, urban areas); and (b) used convenience or non-random sampling to select subjects (e.g., volunteers, clinic or hospital patients, and studies restricted to ethnic minorities, immigrant populations, or non-representative subgroups).

We included studies or surveys that reported the national prevalence of sub-clinical VAD defined as low serum/plasma retinol (ROH) or low retinol binding protein (RBP). Survey results were included regardless of whether ROH or RBP were adjusted for inflammation, but inflammation-adjusted results were prioritized, and were presented if available. Multiple approaches can be used to adjust ROH/RBP results for inflammation, but we did not catalogue the approach used in each survey.

### 2.4. Deficiency and Coverage Definitions

For countries reporting VAD prevalence, we used the WHO guidelines [33] to classify the severity of the public health problem in children 6–59 months, which considers a VA prevalence of 2%–9% a mild public health problem, 10%–19% a moderate public health problem, and ≥20% a severe public health problem. To interpret our findings, we followed the GAVA framework [21], which recommends that countries with VAD prevalence <5% can consider scaling back VAS. Following the approach used in UNICEF’s 2007 progress report [34], we used a coverage of ≥70% as a threshold to identify countries with relatively high VAS coverage.

## 3. Results

### 3.1. VAS Coverage and Overlap with Other VA Programs

As shown in Table 1, the UNICEF’s SOWC VAS database is comprised of 82 countries, of which 77 had VAS coverage results. No coverage results were available for Equatorial Guinea, Kazakhstan, Mexico, Morocco, and Turkmenistan. The coverage of VA supplements ranges from 0% to 99%, and the median coverage is 70%. The coverage of VAS was ≥70% in 38 countries.

Of all 82 countries explored, 54 were implementing at least one other VA program. Forty-one implemented programs mass fortifying vegetable oil, sugar, margarine, or wheat flour with VA, and 33 of these countries have mandatory fortification of at least one food. Vegetable oil fortification programs were the most commonly implemented VA-fortification program, and were conducted in 35 countries. Of the countries explored, provitamin A-biofortified crops have been released in 21 countries, 17 of which were also fortifying a staple food (e.g., vegetable oil, sugar, and wheat flour). Twenty-one countries were implementing MNP programs, twelve of which also implemented fortification and/or biofortification programs.

### 3.2. Recentness of Vitamin A Deficiency Data

**Figure 1:**
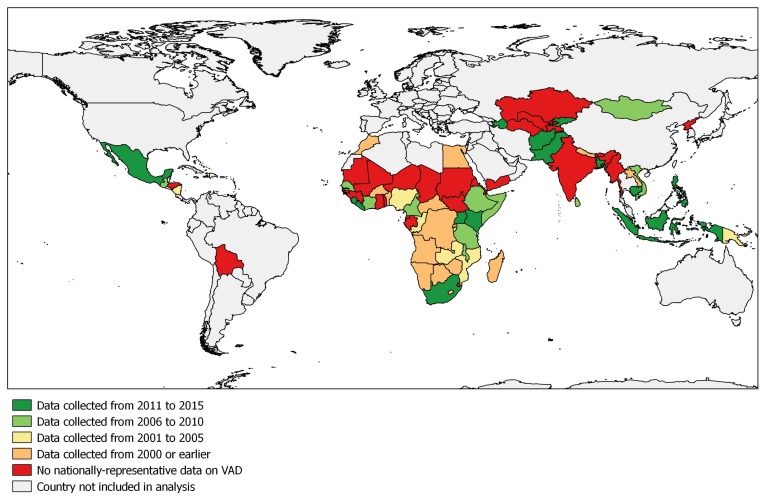
Recentness of nationally-representative data on vitamin A deficiency (VAD).

When examining countries by the recentness of the VAD prevalence data, we found 14 countries with data collected after 2010, 13 countries with data from 2006 to 2010, seven countries with data from 2001 to 2005, and 16 countries with data collected in or prior to 2000. For 32 countries, no nationally-representative data on VAD prevalence could be found. As shown in the map (Figure 1), countries that have not yet collected data on VAD prevalence are predominantly situated in the West African Sahel and Central Asia. In Central Africa, some data have been collected, but are largely outdated. In total, only one-third of the countries explored collected VAD in the past 10 years.

Of the 50 countries that measured VAD prevalence, 35 and 15 countries measured serum/plasma ROH or RBP concentrations, respectively. Twenty surveys accounted for inflammation in some manner, either by excluding all children with inflammation or adjusting ROH or RBP concentrations.

### 3.3. VAS Program and Deficiency Data Comparison

A comparison of VAS coverage and availability of survey data identified 13 countries that have high coverage (i.e., ≥70%) of VA supplements but have never collected national data on VAD prevalence. In Africa, these countries include Burkina Faso, Chad, Congo, Guinea, Guinea Bissau, Mali, Mauritania, Niger, and Sudan. In East and Central Asia, these countries include Myanmar, Democratic People’s Republic of Korea, Tajikistan, and Uzbekistan. Seven countries (Benin, Botswana, Democratic Republic of Congo (DRC), Nepal, Nigeria, Rwanda, and Zambia) had high VAS coverage (i.e., ≥70%) and national VAD data collected more than 10 years ago (i.e., prior to 2006).

Ten countries (i.e., Bolivia, Bhutan, Djibouti, Eritrea, India, Kiribati, Micronesia, Sao Tome and Principe, Swaziland, and Togo) had VAS coverage between 40%–69% but no national VAD data, and four countries had the same VAS coverage but data collected prior to 2006 (i.e., Egypt, Honduras, Lesotho, and Namibia).

In the 34 countries where VAD is a severe public health problem (measured by a national survey in any time period), 19 had VAS coverage ≥70%, whereas 15 countries had moderate to low VAS coverage (<70%). VAD is a moderate public health problem in eight countries, only two of which (Rwanda and Vietnam) have VAS coverage ≥70%. Among the eight countries where VAD is considered mild or not a public health problem (i.e., deficiency <10%), four have VAS coverage ≥70%, including Cambodia, Indonesia, Kyrgyzstan, and Maldives. The above comparisons must be interpreted with caution because for most countries, the year of VAD measurement is different from the year of coverage assessment.

### 3.4. All Programs and Deficiency Data Comparison

Of the 38 countries with VAS coverage ≥70%, 30 implemented at least one other VA program. Of these 30 countries, 16 had two or more (bio-)fortified foods or VA programs (e.g., vegetable oil fortification and MNPs). Most notably, Nigeria has a VAS coverage of 80% and has mandatory vegetable oil fortification and has released biofortified sweet potato, maize, cassava, and plantain varieties. Similarly, Zambia has VAS coverage of 93%; has fortified vegetable oil and sugar; and has released biofortified sweet potato and maize varieties.

Among all countries examined, we found 12 countries (i.e., Bolivia, Burkina Faso, Djibouti, DPR Korea, Ghana, Guinea, Guinea Bissau, India, Mali, Mauritania, Niger, and Togo) that were implementing VAS at the same time as another VA program but had no nationally-representative data on VAD. Fifteen countries (i.e., Angola, Benin, Burundi, DRC, Egypt, Honduras, Lesotho, Madagascar, Mozambique, Nepal, Nicaragua, Nigeria, Rwanda, Zambia, and Zimbabwe) currently implemented VAS with another VA program but only collected national VAD data in or prior to 2005. More than half of the 51 countries that implemented VAS at the same time as another VA program had no VAD data or data collected prior to 2006.

## 4. Discussion

### 4.1. Implications of Outdated/Missing VAD Data

Our study finds that many countries implement VAS and other programs to improve VA status without evidence of the national prevalence and severity of VAD. This has two critical implications. First, it may prevent program planners from: (a) focusing resources on the most vulnerable areas; and (b) from scaling down their programming in areas (e.g., high-income urban areas) where VA-related mortality incidence and prevalence of VAD may be lower. Second, outdated data and the lack of temporal comparisons prevent program planners from understanding trends in VAD. Understanding deficiency trends is particularly important in countries that have scaled up VAS in parallel with other VA interventions (e.g., (bio-)fortification, MNPs).

Monitoring the potential for excessive intakes is also a growing concern where more than one staple food is (bio-)fortified or multiple interventions are occurring [86]. For example, VAD data in West Africa is largely missing or outdated, but VAS and fortification programs have been implemented concurrently since 2006 when a regional vegetable oil fortification project (Tâche d’Huile) was first launched at the country level [87]. By 2013, an estimated 75% of the populations in eight West African countries were consuming fortified vegetable oil [87]. Recent reports from Abidjan, Côte d’Ivoire, found that 97% of vegetable oil was adequately fortified (i.e., 8 μg retinol equivalents/g) and increased the nutrient intake of VA by 27% in children 6–23 months of age [88].

It is important to note, however, that overlapping VA interventions at the country level does not necessarily imply excessive intakes will occur. First, the distribution and consumption of VA (bio-)fortified foods and MNPs may vary at the sub-national level. Second, sub-optimal program implementation may limit the intake of VA. To illustrate, Luthringer et al. [89] examined quality assurance data from 20 national fortification programs and found that more than half of the food samples tested were *inadequately* fortified and did not meet national fortification standards. Despite this review by Luthringer, performance data on VA interventions is often missing or outdated. This hampers the ability of national programmers to determine if vulnerable populations are consuming sufficient levels of VA, and hampers their ability to identify potential excessive VA intake.

### 4.2. Filling the Data Gap

To address the gap in VAD data, national micronutrient surveys (measuring ROH or RBP in children) should be implemented in countries where data are lacking or outdated. In some cases, using more sensitive methodology (e.g., modified relative dose response (MRDR), isotope dilution tests [90]) on randomly selected subsamples may be important, especially if widespread fortification programs are in place.

In 2012, GAVA recommended that program managers prioritize survey results published in the last ten years [21]. This was based on an assumption that the epidemiological landscape of developing countries and degree of introduction of other VA programs had changed since the 1990s, and so the VAD prevalence measured prior to 2000 would not sufficiently reflect the current context. While there is no set interval between surveys that is recommended, data on VA status should be updated regularly, particularly in settings where the consumption of VA-rich foods has changed by the introduction of bio(fortification) programs or improved dietary diversity. Thus, we recommend that VA assessments be conducted at least every ten years to account for changing consumption patterns and the increasing coverage of (bio-)fortified foods.

When filling gaps in VAD data, surveys should also measure the coverage of VA interventions. This ideally includes estimations of additional VA intake originating from (bio-)fortified foods and supplements with VA (e.g., MNPs). As part of a recent survey in Abidjan, Côte d’Ivoire [88], additional intake of VA from fortified vegetable oil was calculated using information of quantities of oil consumed (mL/day) and actual VA levels in oil (μg/L), expressed as percentage of recommended nutrient intake (RNI).

There are, however, challenges to implementing national micronutrient surveys, and these challenges may, in part, explain some of the data gaps observed. Micronutrient surveys are more complex than other commonly-implemented representative surveys (e.g., Demographic and Health Surveys, Multiple Indicator Cluster Surveys, and Standardized Monitoring and Assessment of Relief and Transitions (SMART) surveys) because they require the collection of questionnaire data and blood samples for biomarker analysis. Although some DHS surveys (e.g., Cambodia) have incorporated a micronutrient component, this practice is rare. Micronutrient surveys need a cold chain for blood samples, which requires additional effort and resources and can pose challenges when surveying remote areas. Another challenge to VA status surveys is the fact ROH and RBP are depressed in the presence of inflammation. Thus, inflammation biomarkers, such as C-reactive protein and alpha 1-acid glycoprotein, positive acute phase proteins, should be measured in VAD surveys to adjust ROH or RBP concentrations, and be used to examine inflammation-adjusted and unadjusted VAD prevalence rates. These inflammation markers will assist in interpreting low ROH values [91]. Various adjustment approaches exist [92,93], and there is a growing consensus that VAD should only be estimated once ROH or RBP are adjusted using *both* C-reactive protein and alpha 1-acid glycoprotein. Despite these obstacles, micronutrient surveys have been successfully undertaken in countries with poor road infrastructure, limited electricity networks, and only basic laboratory capacity. While it is difficult to speculate as to why VAD data exist in some countries and not others, a notable pre-requisite for any national VA assessment is demand from national nutrition stakeholders for representative data and available resources.

### 4.3. Alignment of Vitamin A Deficiency Results and Supplementation Programs

The main rationale for implementing VAS programs is to prevent mortality. It is widely agreed that VAD data are needed to better target VAS programs and to justify scaling up/down VAS programs [94].

Our analysis identified only three countries, Nicaragua, Guatemala, and Indonesia, that have VAD prevalence <5%, the threshold recommended by GAVA. Nicaragua and Guatemala mandate that sugar be fortified with VA, and Guatemala also mandates fortification of margarine. The coverage of VAS was reported as 4% in Nicaragua and 19% in Guatemala in 2014. Due to evidence of low VAD prevalence, the Guatemalan government recommended that VAS be scaled back to younger children and no other foods be fortified. Furthermore, due to concern of excessive VA intakes in Guatemala from fortified sugar, the government is considering lowering the amount of VA added to 7 mg/kg sugar [95]. In Indonesia, the very low VAD prevalence observed by the 2011 national survey [53] is contrasted by a 2011 evaluation of fortified vegetable oil in West Java [96] that found VAD prevalence between 10% and 18% in preschool children. Though the evaluation was only conducted in 24 villages, it illustrates that nationally-aggregated VAD results may mask sub-national variations. In the case of this evaluation, consumption of VA-fortified vegetable oil for one year markedly reduced VAD in preschool children, particularly in children 24–59 months of age where VAD was reduced from 10% at baseline to <1% at endline. As the fortification of vegetable oil with VA was made mandatory in Indonesia in 2013 and was in full effect by 2015 [97] more recent studies in Indonesia examining VAD, and potentially excessive intakes of VA, should be considered.

Kyrgyzstan offers an example of where national VAD results influenced VAS program policy. VAD was estimated at 4.2% in 2009, and since 2010, the VAS program has been replaced by national and universal distribution of an MNP that contains VA and other micronutrients [98,99]. However, a national follow-up survey in 2013 found that VAD prevalence was 7.8% [55].

In addition, a national VA survey should also be conducted in Zambia, as extensive and recent sub-national data suggest that children may consume excess amounts of VA [100]. Two recent studies from Central and Eastern Zambia found that a much lower percentage of children had inadequate liver reserves assessed with the MRDR test [100,101]. Another study in Eastern Zambia found that a large proportion of children 5–7 years of age were experiencing hypervitaminosis A, assessed with ROH isotope dilution [102], and documented hypercarotenodermia during mango season [103], likely due in part to wide-scale sugar fortification on top of a traditional diet high in provitamin A carotenoids [100]. Another study in Central Zambia found that serum ROH concentrations in children 4 to 8 years old did not respond to an intervention with provitamin A biofortified maize [104]. The author’s conclusion was that the children were relatively VA adequate at baseline [104].

### 4.4. Global Debate and Engagement

In the international peer-reviewed literature, the debate on the appropriateness of VAS programs focuses primarily, but not exclusively, on results from clinical trials. This debate is important, and as many researchers rightly point out, there is consistent evidence that VAS programs can reduce mortality and morbidity. We believe that the evidence is underpinning VAS and clearly demonstrates its utility. However, our review suggests that country-level data on VAD are sorely needed, and that several countries implement multiple VA interventions without a current understanding of national VAD prevalence.

Properly done surveys yielding new VAD data will likely have programmatic implications. These surveys may identify specific areas where VAD is rare and the VAS program should be scaled back. In other cases, survey data could suggest that VAS programs should be implemented in parallel with other VA programs (e.g., (bio-)fortification, and MNPs) to reduce mortality while sustainably improving VA status. All that said, this type of speculation is futile without good representative data.

More country level data are needed to make evidence-based decisions, and international agencies that fund VAS programs must support national stakeholders to fill the data gap. While VAD prevalence estimates at a national level is the starting point for a national dialogue, program planners should also examine the sub-national pattern of VAD to decide where and how to scale up/back their VAS program [21]. While debates about efficacy and effectiveness are important, they do little to help national programmers make decisions and, as such, we see this review as a contribution to focus the discussion away from global extrapolations to country-specific discussions.

Lastly, in addition to regularly updated documentation of the VAD prevalence of various population groups, the WHO database can be enhanced by a complementary repository of micronutrient survey reports/publications. Unfortunately, many findings from national micronutrient reports are not subsequently published online or in indexed journals that can be searched in perpetuity.

### 4.5. Limitations

Our study examined only national-level data, and thus data from sub-national but representative studies was not included. Furthermore, our study is limited to publicly available data. Thus, it is possible that national policy makers in some countries included in our analysis have sufficient data on VAD that was missed by our search criteria. Moreover, during our search, the authors noted that some surveys were currently being implemented or results were not yet publicly released, such as national surveys in India, Myanmar, Nepal, and Ethiopia. In addition, this inventory only presented the prevalence of VAD as reported by survey reports and publications, and did not catalogue the various blood collection methods used, analysis technique used, or if and how ROH or RBP concentrations were adjusted for inflammation.

We reported the most recent VAS coverage as presented in UNICEF’s SOWC VAS coverage database, but in some cases, this coverage may lead to misinterpretations of program coverage and performance. For example, Sierra Leone and Bangladesh had a two-dose coverage of 8% and 0% in 2014, but a coverage of >90% in previous years [105]. This illustrates that implementation irregularities in VAS programs continue to exist, despite being a well-established, scaled-up intervention in many countries. In addition, as we only examined the 82 countries included as part UNICEF’s VAS coverage database, our analysis likely excluded some countries where small scale VAS programs are conducted.

Lastly, although we present the fortification, biofortification, and MNP programs implemented in each country, no comprehensive source of coverage results for these foods could be identified. The gap in information about VA program coverage has been noted elsewhere, and has been identified as a key challenge to identifying if children are being exposed to any or multiple VA interventions [106]. Biofortified staple crops are relatively new in the scheme of nutritional interventions. In Zambia by 2015, seeds of one biofortified maize variety had gained only 1% of the market [107]. Lastly, our analysis did not include fortified blended flours (e.g., corn–soy blend plus), lipid-based nutrient supplements, or fortified complementary food products (e.g., porridges).

## 5. Conclusions

VAS programs are implemented in ~80 low- and middle-income countries. Despite the widespread implementation of this type of program, many countries have either no data or outdated data on VAD prevalence. Changing consumption patterns and the expansion of VA (bio-)fortified foods warrants that countries, particularly those implementing VAS programs, measure VA status in children at least every 10 years, or when coverage and consumption data indicate that a shift in VAD prevalence may have occurred. At the international level, UN agencies and non-governmental organizations should provide technical support to undertake national micronutrient surveys. At the national level, program planners should make evidence-based decisions, and if biochemical data of VA status are not sufficient, it should be collected before changes to programs are made. Regarding the global debate about VAS programs, we found that a few countries have already begun scaling down VAS programs, and agree that it is an option in countries where VAD prevalence has been repeatedly shown to be low. Where VAD is a public health problem, however, VAS programs can and should be implemented in parallel with other programs to improve VA intake and reduce under-five mortality. However, given the lack of VAD data at the country level, scaling down VAS programs may be a premature undertaking. While the global debate about VAS is important, more attention should be directed towards the situation in individual countries where programmatic decisions are made.

## Figures and Tables

**Table 1:** The most recent estimates of VAS coverage, presence of other VA programs, and VAD prevalence in countries in UNICEF’s SOWC VAS coverage database ^1^.

Countries and Territories	Coverage (%) of VAS Program	Year of Coverage Estimate	VA Fortification, Biofortification, and MNP Programs ^2^	Year of Most Recent Nationally-Representative VAD Survey	Biomarker ^3^	VAD Prevalence (%) ^4^	Severity of VAD	Source
Afghanistan	95	2014	fVO ^(v)^, fW ^(v)^	2013	ROH	*50.4*	Severe	[35]
Angola	6	2014	bSP ^(v)^	1999	ROH	64.3	Severe	[36] *
Azerbaijan	58	2014		2013	RBP	*8.0*	Mild	[37]
Bangladesh	0	2014	fVO ^(m)^, fW ^(v)^, MNP ^(v)^	2011	ROH	*20.5*	Severe	[38]
Benin	99	2014	fVO ^(m)^	1999	ROH	82.0	Severe	[39] *
Bhutan	45	2013						
Bolivia	40	2013	fVO ^(m)^, MNP ^(v)^					
Botswana	70	2014		1994	ROH	35.4	Severe	[40] *
Burkina Faso	98	2014	fVO ^(m)^, bSP ^(v)^					
Burundi	68	2014	fVO ^(v)^, bP ^(v)^	2005	ROH	27.9	Severe	[41]
Cambodia	71	2014	fVO ^(v)^, MNP ^(v)^	2014	RBP	9.2	Mild	[42]
Cameroon	96	2014	fVO ^(m)^, bP ^(v)^	2009	RBP	*35.0* ^††^	Severe	[43]
Central African Rep	34	2014		1999	ROH	68.2	Severe	[44] *
Chad	96	2014						
Comoros	14	2014						
Congo	99	2014						
Côte d’Ivoire	99	2014	fVO ^(m)^	2007	RBP	*24.1*	Severe	[45]
DRC	99	2014	bC ^(v)^, bP ^(v)^	1998/99	-ROH	61.1	Severe	[46]
Djibouti	66	2013	MNP ^(v)^					
Egypt	68	2008	fVO ^(v)^	1995	ROH	11.9	Moderate	[47] *
Equatorial Guinea								
Eritrea	49	2014						
Ethiopia	71	2014	bSP ^(v)^	2006 ^‡^	ROH	37.7	Severe	[48]
Gabon	2	2012						
Gambia	27	2014		1999	ROH	64.0	Severe	[49] *
Ghana	23	2014	fVO ^(m)^, fW ^(m)^, bSP ^(v)^, bM ^(v)^					
Guatemala	19	2014	fMG ^(m)^, fS ^(m)^, MNP ^(v)^	2009/10	ROH	0.3	None	[50]
Guinea	99	2012	fVO ^(m)^					
Guinea-Bissau	98	2014	fVO ^(m)^					
Haiti	30	2014	MNP ^(v)^	2005	ROH	32.0	Severe	[51] *
Honduras	40	2005	fMG ^(m)^, fS ^(m)^	1996	ROH	13.8	Moderate	[52]
India	61	2014	fVO ^(v)^, bSP ^(v)^					
Indonesia	84	2014	fVO ^(m)^, fW ^(m)^	2011	ROH	<1	None	[53]
Kazakhstan								
Kenya	28	2014	fVO ^(m)^, fS ^(v)^, bSP ^(v)^	2012	RBP	*9.2*	Mild	[54]
Kiribati	54	2006						
Kyrgyzstan	97	2010	MNP ^(v)^	2013	RBP	*7.8*	Mild	[55]
Laos	89	2014		2000	ROH	44.7	Severe	[56]
Lesotho	67	2014	fW ^(v)^	1993	ROH	78.0	Severe	[57] *
Liberia	0	2014	fVO ^(m)^, fS ^(m)^	2011	RBP	*13.2*	Moderate	[58]
Madagascar	99	2014	bSP ^(v)^, MNP ^(v)^	2000	ROH	42.1	Severe	[59]
Malawi	41	2014	fVO ^(m)^, fS ^(m)^, bSP ^(v)^, bC ^(v)^, MNP ^(v)^	2009	RBP	*22.0* ^††^	Severe	[60]
Maldives	76	2013		2007	ROH	5.1	Mild	[61]
Mali	98	2013	fVO ^(v)^, bM ^(v)^					
Marshall Islands	39	2007		1995	ROH	59.9	Severe	[62]
Mauritania	89	2014	fVO ^(m)^					
Mexico			fMG ^(m)^, MNP ^(v)^	2011/12	ROH	15.7	Moderate	[63]
Micronesia	68	2006						
Mongolia	79	2014	MNP ^(v)^	2010	RBP	32.4	Severe	[64]
Morocco			fVO ^(m)^	1996	ROH	40.4	Severe	[65]
Mozambique	99	2014	fVO ^(m)^, bSP ^(v)^, MNP ^(v)^	2002	ROH	68.8	Severe	[66]
Myanmar	94	2014	MNP ^(v)^					
Namibia	62	2013		1992	ROH	23.5	Severe	[67] *
Nepal	85	2014	MNP ^(v)^	1998 ^‡^	ROH	32.3	Severe	[68]
Nicaragua	4	2014	fS ^(m)^, MNP ^(v)^	2004	ROH	2.1	Mild	[69]
Niger	95	2014	fVO ^(m)^, fS ^(m)^, bSP ^(v)^					
Nigeria	80	2014	fVO ^(m)^, fW ^(m)^, bSP ^(v)^, bM ^(v)^, bC ^(v)^, bP ^(v)^	2001	ROH	29.5	Severe	[70]
DPR Korea	99	2014	MNP ^(v)^					
Pakistan	96	2014	fVO ^(m)^	2011	ROH	*54.0*	Severe	[71]
Papua New Guinea	15	2012		2005	RBP	*15.7*	Moderate	[72]
Philippines	83	2014	fVO ^(m)^, fW ^(m)^, MNP ^(v)^	2013	ROH	*20.4*	Severe	[73]
Rwanda	95	2014	fVO ^(m)^, fS ^(m)^, bSP ^(v)^, MNP ^(v)^	1996	ROH	6.4	Moderate	[74]
Sao Tome and Principe	56	2013						
Senegal	89	2014	fVO ^(m)^, bSP ^(v)^, MNP ^(v)^	2010	ROH	*17.7*	Severe	[75]
Sierra Leone	8	2014	fVO ^(m)^, bC ^(v)^	2013	RBP	*17.4*	Moderate	[76]
Somalia	30	2014	MNP ^(v)^	2009	RBP	*33.3* ^††^	Severe	[77]
South Africa	42	2013	fMG ^(v)^, fW ^(m)^	2012	ROH	43.6	Severe	[78]
South Sudan	18	2014						
Sri Lanka	72	2014	MNP ^(v)^	2006	ROH	29.3	Severe	[79]
Sudan	99	2014						
Swaziland	43	2014						
Tajikistan	99	2014						
Tanzania	88	2014	fVO ^(m)^, fS ^(m)^, bSP ^(v)^, MNP ^(v)^	2010	RBP	*33.0* ^††^	Severe	[80]
Timor-Leste	40	2013		2013	RBP	*9.7*	Mild	[81]
Turkmenistan								
Togo	61	2013	fVO ^(m)^					
Uganda	66	2014	fVO ^(m)^, fW ^(m)^, bSP ^(v)^	2011	RBP	*32.6* ^††^	Severe	[82]
Uzbekistan	99	2014						
Vietnam	94	2014	fVO ^(m)^	2010	ROH	*10.1*	Moderate	[83]
Yemen	7	2014						
Zambia	93	2013	fVO ^(v)^, fS ^(m)^, bSP ^(v)^, bM ^(v)^	2003	ROH	54.1	Severe	[84]
Zimbabwe	32	2014	fVO ^(v)^	1999	ROH	35.8	Severe	[85] *

^1^ VA, vitamin A; VAD, vitamin A deficiency; VAS, vitamin A supplementation; UNICEF, United Nations Children’s Fund; SOWC, State of the World’s Children.^2^ fVO = fortified vegetable oil, fMG = fortified margarine, fS = fortified sugar, fW = fortified wheat flour; bSP = biofortified sweet potato, bM = biofortified maize, bC = biofortified cassava, bP = biofortified plantain/banana; MNP = micronutrient powders. ^(m)^ = mandatory program, ^(v)^ = voluntary program; ^3^ ROH, serum/plasma retinol; RBP, retinol-binding protein. ^4^ VAD prevalence measured as proportion of children with ROH or RBP concentrations <0.7 μmol/L, unless noted otherwise. Prevalences in *italics* indicate that prevalence calculation accounted for inflammation in some manner (e.g., adjusting ROH or RBP concentrations, excluding children with any inflammation, etc); * Data source taken from the World Health Organization Global Database on Vitamin A Deficiency; ^††^ VAD prevalence measured as proportion of children with RBP <0.825 μmol/L in Uganda, Somalia, and Tanzania; <0.83 μmol/L in Cameroon, <0.78 µmol/L in Malawi; ^‡^ A more recent survey was conducted, but the results were not publically available at the time of writing this manuscript.
